# Quantitative DCE-MRI demonstrates increased blood perfusion in Hoffa’s fat pad signal abnormalities in knee osteoarthritis, but not in patellofemoral pain

**DOI:** 10.1007/s00330-020-06671-6

**Published:** 2020-02-17

**Authors:** Bas A. de Vries, Rianne A. van der Heijden, Dirk H. J. Poot, Marienke van Middelkoop, Duncan E. Meuffels, Gabriel P. Krestin, Edwin H. G. Oei

**Affiliations:** 1 Department of Radiology & Nuclear Medicine, Erasmus University Medical Center Rotterdam, Rotterdam, The Netherlands; 2 Department of Medical Informatics, Erasmus University Medical Center Rotterdam, Rotterdam, The Netherlands; 3 Department of General Practice, Erasmus University Medical Center Rotterdam, Rotterdam, The Netherlands; 4 Department of Orthopedic Surgery, Erasmus University Medical Center Rotterdam, Rotterdam, The Netherlands

**Keywords:** Osteoarthritis, knee, Patellofemoral pain syndrome, Inflammation, Magnetic resonance imaging, Synovitis

## Abstract

**Objective:**

Infrapatellar fat pad (IPFP) fat-suppressed T2 (T2_FS_) hyperintense regions on MRI are an important imaging feature of knee osteoarthritis (OA) and are thought to represent inflammation. These regions are also common in non-OA subjects, and may not always be linked to inflammation. Our aim was to evaluate quantitative blood perfusion parameters, as surrogate measure of inflammation, within T2_FS_-hyperintense regions in patients with OA, with patellofemoral pain (PFP) (supposed OA precursor), and control subjects.

**Methods:**

Twenty-two knee OA patients, 35 PFP patients and 43 healthy controls were included and underwent MRI, comprising T2 and DCE-MRI sequences. T2_FS_-hyperintense IPFP regions were delineated and a reference region was drawn in adjacent IPFP tissue with normal signal intensity. After fitting the extended Tofts pharmacokinetic model, quantitative DCE-MRI perfusion parameters were compared between the two regions within subjects in each subgroup, using a paired Wilcoxon signed-rank test.

**Results:**

T2_FS_-hyperintense IPFP regions were present in 16 of 22 (73%) OA patients, 13 of 35 (37%) PFP patients, and 14 of 43 (33%) controls. DCE-MRI perfusion parameters were significantly different between regions with and without a T2_FS_-hyperintense signal in OA patients, demonstrating higher Ktrans compared to normal IFPF tissue (0.039 min^−1^ versus 0.025 min^−1^, *p* = 0.017) and higher Ve (0.157 versus 0.119, *p* = 0.010). For PFP patients and controls no significant differences were found.

**Conclusions:**

IPFP T2_FS_-hyperintense regions are associated with higher perfusion in knee OA patients in contrast to identically appearing regions in PFP patients and controls, pointing towards an inflammatory pathogenesis in OA only.

**Key Points:**

• *Morphologically identical appearing T2*_*FS*_*-hyperintense infrapatellar fat pad regions show different perfusion in healthy subjects, subjects with patellofemoral pain, and subjects with knee osteoarthritis.*

• *Elevated DCE-MRI perfusion parameters within T2*_*FS*_*-hyperintense infrapatellar fat pad regions in patients with osteoarthritis suggest an inflammatory pathogenesis in osteoarthritis, but not in patellofemoral pain and healthy subjects.*

## Introduction

The infrapatellar fat pad (IPFP), also known as “Hoffa’s fat pad,” is an intracapsular, extra-synovial structure in the anterior knee joint and is one of several fat pads of the knee. This structure has been proposed as possible source of knee pain in patients suffering from osteoarthritis (OA) and from the supposed precursor of knee OA: patellofemoral pain (PFP) [1–6]. In OA research, the MRI Osteoarthritis Knee Score (MOAKS) is one of the most commonly used scoring systems for knee OA on MRI [7]. In this method, the presence and size of hyperintense signal within the IPFP is scored on unenhanced fat-suppressed MR images. These hyperintense lesions are thought to be a manifestation of knee inflammation and are therefore classified as Hoffa synovitis [7]. Moreover, multiple studies have emphasized the importance of T2_FS_-hyperintense IPFP regions as a precursor for structural knee OA [8–12].

A recent study that included patients with PFP and healthy controls subjects showed that T2_FS_-hyperintense regions in the IPFP are rather common [13]. The question arises whether this identically appearing feature, commonly encountered in daily clinical practice and considered an “early OA” feature, has a different pathophysiology across populations. A hyperintense T2_FS_ signal may be caused by edema due to inflammatory induced vasodilatation, but a prior study by Roemer et al suggested that this feature may not always be linked to inflammation [14]. Other causative effects of a fluid signal might be edema due to mechanical friction/impingement or increased vascularity due to neo-angiogenesis, necrosis, or cellular infiltration [15, 16].

Dynamic contrast-enhanced (DCE) MRI enables further evaluation of the pathophysiology of IPFP T2_FS_-hyperintense lesions. Fitting a pharmacokinetic model to the DCE-MRI data enables quantitative surrogate measurement of physiological parameters such as blood flow, blood volume, and extravascular permeability [17]. Increased blood perfusion, evaluated by DCE-MRI, has been considered a surrogate measure of inflammation for a variety of musculoskeletal tissues [18–23]. To the best of our knowledge, the only research with regard to DCE-MRI in the IPFP was performed by Ballegaard et al [23]. They studied obese patients with knee OA using a heuristic DCE-MRI analysis approach and found a positive correlation between knee pain and their DCE-MRI-derived inflammation marker and between knee pain and Hoffa-synovitis assessed by MOAKS, thereby stipulating the importance of the IPFP and the potential of DCE-MRI as a biomarker of inflammation [23]. So far, DCE-MRI has not been applied for studying the pathogenesis of T2_FS_-hyperintense IPFP regions in an OA and non-OA population.

Therefore, the aim of this study was to evaluate differences in quantitative DCE-MRI blood perfusion parameters between a T2_FS_-hyperintense region of the IPFP and adjacent IPFP tissue with normal signal intensity within patients with knee OA, patients with PFP and healthy control subjects. Our hypothesis was that T2_FS_-hyperintense IPFP regions demonstrate different DCE-MRI perfusion parameters in patients with OA, patients with PFP, and healthy control subjects, with the highest degree of perfusion expected in patients with OA.

## Materials and methods

### Study population

In the current study, we analyzed data from two previous studies in order to include both patients with OA and patients with PFP, the supposed precursor of OA. In the first study, patients with unicompartmental radiographic knee OA with a severity of KL (Kellgren and Lawrence [24]) grade 2 and higher, aged 52 to 75 years, scheduled to undergo knee replacement surgery, were included. Patients were excluded when the glomerular filtration rate was < 60 mL/min. In the second study, healthy controls and patients with PFP, aged between 18 and 40 years, were included. Patients were excluded if they had other defined pathological conditions of the knee such as patellar tendinopathy or osteoarthritis, if the onset of PFP occurred after trauma, if they had previous knee injuries or surgery or previous episodes of PFP that occurred more than 2 years ago, or if they had contraindications for MRI scanning with contrast administration. Patients of both studies were included between 2013 and 2017 at the Erasmus University Medical Center Rotterdam (Rotterdam, The Netherlands); details of each study have been published elsewhere [13, 25]. Both studies were approved by the institutional review board of Erasmus MC and written informed consent was obtained from all subjects.

### MR imaging acquisition

In both studies, all subjects underwent MRI using the same MRI scanner and an identical MRI protocol. Multisequence MRI was performed using a 3-T MRI system (Discovery MR750, General Electric Healthcare) and a dedicated 8-channel transmit/receive knee coil (Invivo). DCE-MRI was acquired in the sagittal plane, using a fat-suppressed 3D fast spoiled gradient echo (FSPGR) sequence with 35 phases of 10 s. Contrast agent 0.2 mmol/kg gadopentetate dimeglumine (Magnevist, Bayer) was administrated intravenously with a power injector at a rate of 2 ml/s started after the first phase and followed by a saline flush. The field-of-view (FOV) was 22 × 22 cm, with an in-plane resolution of 0.85 × 1.20 mm and 5-mm slice thickness, and a flip angle of 30° and repetition time of 9.3 ms was used. T2 mapping was performed using a iMSDE prepared 3D fast spin echo (FSE) sequence with a FOV of 15 × 15 cm, 3-mm slice thickness, and an in-plane resolution of 0.52 × 0.78 mm, using 5 different echo times in the preparation module (3.1, 13.4, 27.0, 40.7, 68.1 ms). The protocol also included a fat-suppressed sagittal T2-weighted FSE sequence with a FOV of 15 × 15 cm, 3-mm slice thickness, and an in-plane resolution of 0.36 × 0.59 mm.

### Image analysis

**Fig. 1:**
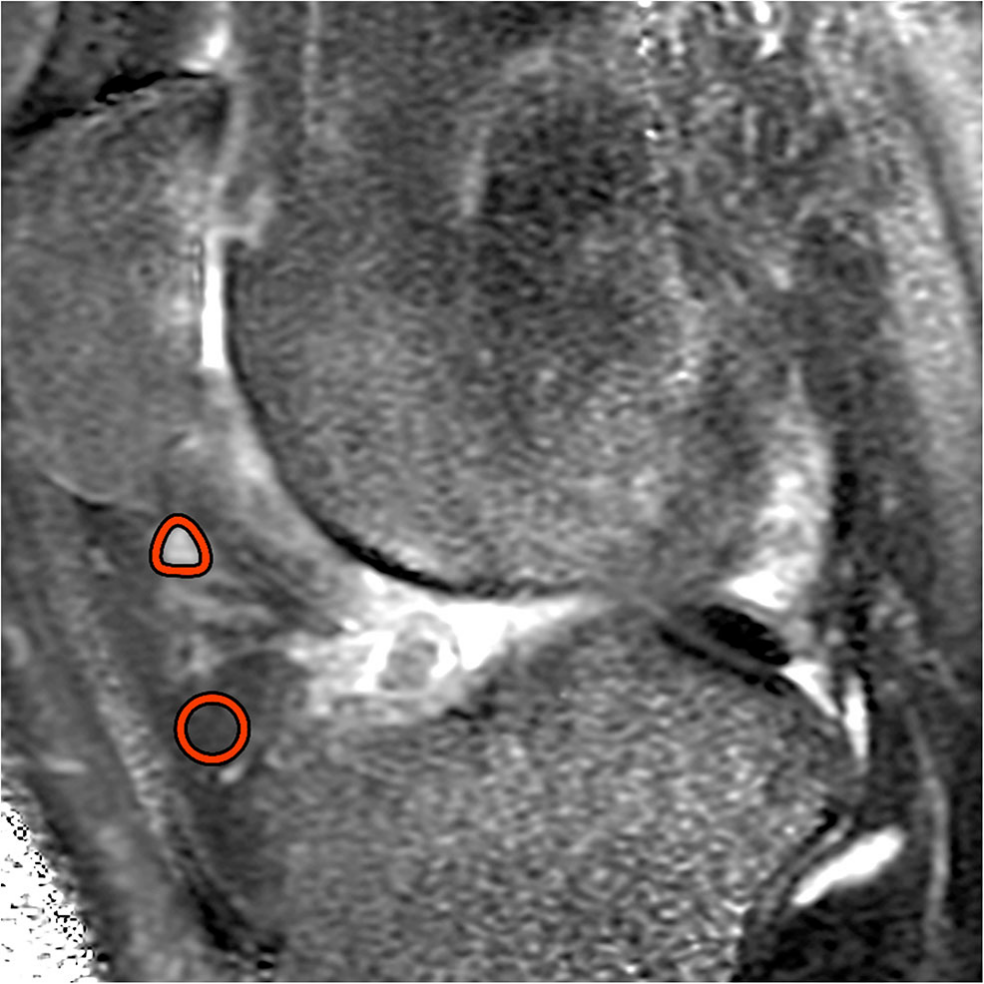
Two ROIs were drawn in the IPFP, one within the T2_FS_-hyperintense region and the second in an adjacent area without T2-hyperintensity

To correct for patient movement, all 35 time points of the DCE-MRI were registered using an automated rigid body registration with Elastix [26]. We first assessed the fat-saturated T2-weighted images for the presence of T2_FS_-hyperintense regions in the IPFP. Subsequently, detected T2_FS_-hyperintense regions were delineated on the quantitative T2 maps. The delineation of ROIs was performed on T2 maps as these images were scanned in the same part of the scan session as the DCE-MRI, in contrast to the T2_FS_-weighted images, and thus the regions of interest (ROIs) could be copied to the DCE-maps. ROIs were placed within the borders of the hyperintense regions using the Horos software package (Horosproject.org). When multiple hyperintense regions were found in the IPFP, the ROI was placed in only one, the largest region. Two ROIs were drawn in the IPFP, one within the T2_FS_-hyperintense region and the second in an adjacent area without T2-hyperintensity (Fig. 1). All ROIs were drawn by a researcher with a technical medical degree and more than three years of experience in musculoskeletal imaging research (B.d.V.). The same ROIs from the T2-maps were copied to the registered DCE-MR images to extract quantitative DCE measures from the same regions. These quantitative DCE parameters (Ktrans, Kep, Ve, Vp) were calculated by fitting the extended Tofts pharmacokinetic model to the DCE-MRI data, using the DCETool in Horos [27, 28]. Subsequently, mean T2 value and mean perfusion parameter values of the ROIs were calculated. The Tofts pharmacokinetic model is widely used for this purpose and has shown to be the most accurate model for patellar bone [29]. For highly vascularized tissues, like the IPFP, the extended Tofts pharmacokinetic model is more suitable due to the addition of the vascular term Vp; therefore, in this study, we used the extended Tofts pharmacokinetic model [30]. Ktrans reflects the volume transfer constant into the tissue compartment, Kep describes the rate constant back into the vascular component, Ve is the extravascular extracellular space, and Vp is the vascular fraction of the region [31]. The arterial input function (AIF) was estimated using a ROI in the popliteal artery. All fitted AIFs were visually checked.

### Statistical analysis

The image analyses result in a mean value for the T2 and perfusion parameters within each region. For each region, the median T2 and perfusion parameters over all subjects in a certain group were calculated, as well as the interquartile range (IQR) as a measure of variability. Since all DCE-MRI variables showed a non-normal distribution, using the Shapiro-Wilk test, a paired Wilcoxon signed-rank test was used to compare perfusion parameter values of the T2_FS_-hyperintense region with the adjacent region with normal signal intensity within the different subject groups. A Mann-Whitney *U* test was used to evaluate the location distribution of T2_FS_-hyperintense IPFP regions over the groups as well as differences in DCE-MRI perfusion parameters of a central versus a peripheral T2_FS_-hyperintense region. Statistical analysis was performed using SPSS v25 (IBM). *P* values < 0.05 were considered to be statistically significant.

## Results

In total, 100 participants were included from both studies: 22 patients with knee OA, 35 patients with PFP, and 43 healthy controls. The mean BMI was higher in the OA group (30.6 kg/m^2^) in comparison to the PFP and the control group with a mean BMI of 24.6 and 22.3 kg/m^2^, respectively. The Knee Injury and Osteoarthritis Outcome Score (KOOS) indicated that pain symptoms were most severe in the OA group. Characteristics of all participants are shown in Table 1.

**Table 1:** Characteristics of participants with T2 regions within IPFP

Groups Parameter	OA Patients *N* = 16	PFP patients *N* = 13	Controls *N* = 14	Total *N* = 43
Sex male (%)	5 (31%)	8 (62%)	7 (50%)	20 (47%)
Age in years	63.3 ± 6.3^a^	27.0 ± 5.6^a^	25.8 ± 4.4^a^	29.6^b^ [24.0–60.0]
BMI in kg/m^2^	30.6 ± 5.2^a^	24.6 ± 3.5^a^	22.3 ± 2.2^a^	24.3^b^ [21.9–29.1]
KOOS pain subscale	40.5 ± 11.0^a^	71.6 ± 17.9^a^	100.0^b^ [100.0–100.0]	66.7^b^ [44.4–100.0]

*SD*: standard deviation, *IQR*: interquartile range

^a^Mean ± SD

^b^Median [IQR]

T2_FS_-hyperintense IPFP regions were present in 43 subjects. The prevalence of the T2_FS_-hyperintense IPFP regions was different between the groups: 16 of 22 (73%) knee OA patients, 13 of 35 (37%) PFP patients, and 14 of 43 (33%) controls. Of the 16 knee OA patients, three had a radiographic OA severity of Kellgren and Lawrence grade 2, eight had KL grade 3, and five patients had KL grade 4.

The median T2 value in IPFP tissue without T2-hyperintensity was 36.4, 33.9, and 32.7 ms in OA patients, PFP patients, and controls, respectively. For the T2_FS_-hyperintense regions, these values were 61.4, 52.3, and 53.7 ms, respectively (Table 2).

**Table 2:** T2 and DCE-MRI perfusion parameters in the IPFP. *p* values of the difference between T2-hyperintense region and tissue with normal signal intensity are reported. *IQR* = interquartile range

	T2 relaxation time (ms)		Ktrans × 1000 (min^−1^)			Kep × 1000 (min^−1^)			Ve × 1000 (unit-less)			Vp × 1000 (unit-less)		
	Median	IQR	Median	IQR	*p* value^†^	Median	IQR	*p* value^†^	Median	IQR	*p* value^†^	Median	IQR	*p* value^†^
OA patients														
T2-hyperintense region	61.40	31.27	39.03	65.79	0.017*	197.57	198.66	0.079	157.19	259.45	0.010*	2.09	6.15	0.363
Normal signal intensity	36.39	6.15	24.73	22.76		163.49	131.83		119.18	151.43		1.03	5.98	
PFP patients														
T2-hyperintense region	52.30	10.66	11.07	13.49	0.552	173.41	198.61	0.552	143.00	128.37	0.917	0.22	0.52	0.477
Normal signal intensity	33.85	4.62	13.61	10.09		112.86	181.96		143.95	125.33		0.11	0.39	
Controls														
T2-hyperintense region	53.67	15.49	9.84	17.28	0.363	91.00	97.38	0.778	160.62	255.09	0.510	0.13	0.70	0.075
Normal signal intensity	32.71	4.74	14.36	23.02		122.28	117.59		181.53	116.12		0.01	0.18	

^†^Wilcoxon signed-rank test

* *p* values < 0.05

Most hyperintense regions were located centrally (*n* = 30) in the IPFP whereas 13 were located more peripherally. We observed no significant difference in location distribution between groups as well as no difference in all DCE-MRI perfusion parameters of a central versus a peripheral T2_FS_ hyperintense region.

**Fig. 2:**
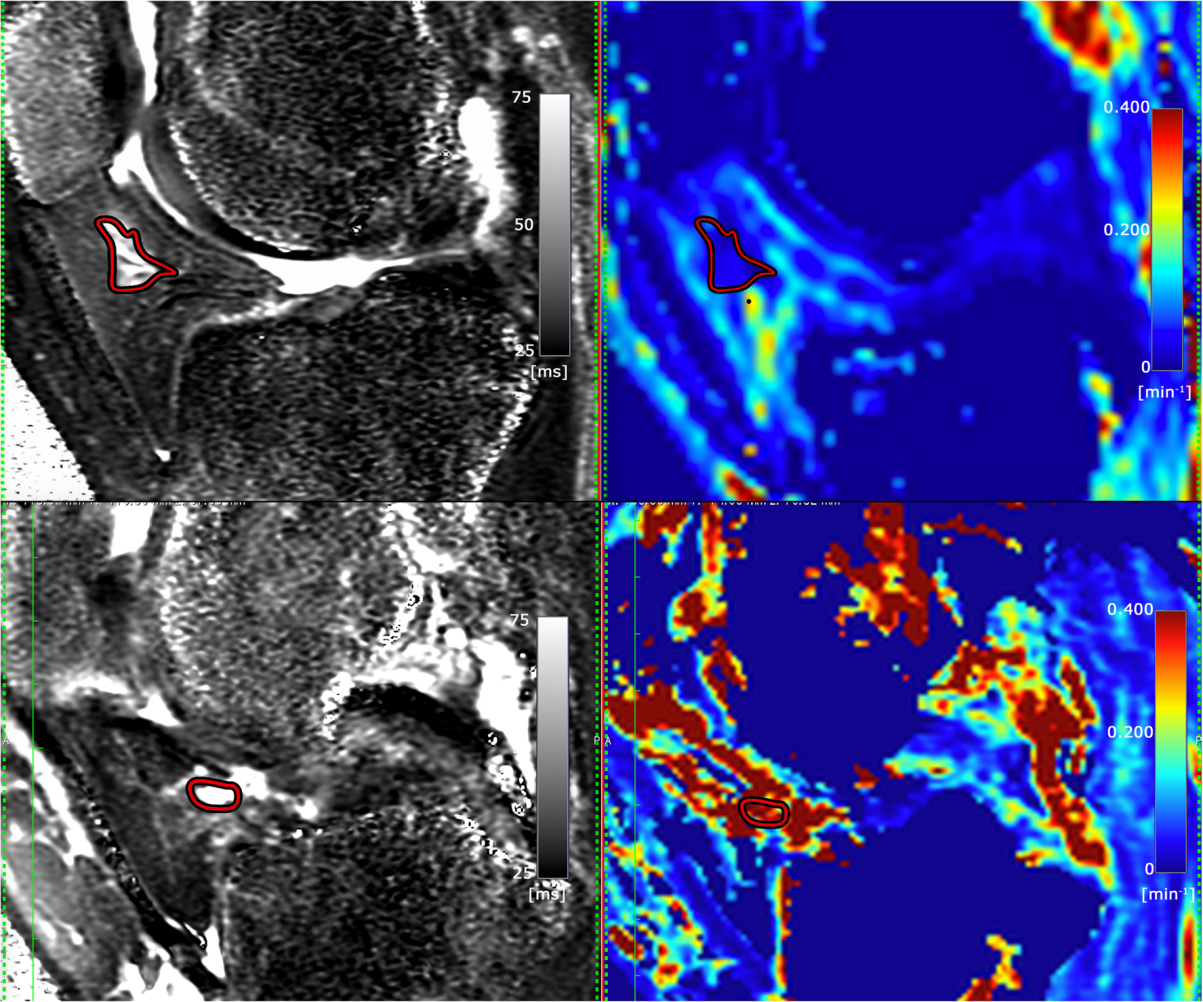
Delineated T2-hyperintense region within IPFP on T2 map (left) and corresponding Ktrans map (values in 1/min) (right) in patient with PFP (upper row) and patient with OA (lower row). Higher values of Ktrans are depicted in red

In knee OA patients, the T2_FS_-hyperintense IPFP regions demonstrated significantly higher values of Ktrans (see Fig. 2) and Ve compared to tissue with normal signal intensity (0.039 min^−1^ vs. 0.025 min^−1^ for Ktrans and 0.157 vs. 0.119 for Ve). Kep and Vp were higher within T2_FS_-hyperintense lesions compared to tissue with normal signal intensity (median Kep 197.57 vs. 163.49 and median Vp 2.09 vs. 1.03, respectively). However, these differences were not statistically significant for both Kep (*p* = 0.079) and Vp (*p* = 0.363). In both controls and PFP patients, all DCE-MRI perfusion parameters were not significantly different between IPFP tissue with and without a T2_FS_-hyperintensity. In PFP-patients, a Ktrans of 0.014 min^−1^ and Kep 0.113 min^−1^ in IPFP tissue with normal signal intensity and a Ktrans of 0.011 min^−1^ and Kep 0.173 min^−1^ in tissue with T2_FS_-hyperintensity was found. In controls, the median Ktrans was 0.014 min^−1^ and median Kep was 0.122 min^−1^ in IPFP tissue with normal signal intensity and in tissue with T2_FS_-hyperintensity these values were 0.010 and 0.091 min^−1^, respectively. Moreover, all DCE-MRI perfusion parameters were higher in both the hyperintense lesions and normal IPFP tissue in the OA group. All DCE-MRI results are shown in Table 2.

## Discussion

In this study, quantitative DCE-MRI perfusion parameters were measured within T2_FS_-hyperintense regions and adjacent IPFP tissue with normal signal intensity of patients with knee OA and patients with PFP and in healthy controls. Our hypothesis was that identically appearing T2_FS_-hyperintense IPFP regions in patients with OA, PFP, and control subjects demonstrate different degrees of increased perfusion measured with quantitative DCE-MRI compared to adjacent IPFP tissue with normal signal intensity. We expected the highest perfusion in patients with OA, in which term Hoffa synovitis has been coined to describe such regions. Indeed, we found that T2_FS_-hyperintense regions showed significantly increased perfusion compared to adjacent IPFP tissue with normal signal intensity in OA patients only, in contrast to both patients with PFP and healthy controls. This finding suggests an inflammatory pathogenesis of such regions in OA patients, but not in patients with PFP and healthy control subjects. Our observation that knee OA patients demonstrated, in general, higher DCE-MRI perfusion parameters than PFP patients and healthy controls, irrespective of the presence of a T2_FS_-hyperintense region, also indicate that the entire IPFP may be affected by inflammation in OA and possibly also by neo-angiogenesis, based on the elevated Vp, which represents the vascular fraction within the ROI. Our observation of this phenomenon in the IPFP is of interest, as from previous literature it is known that OA is not a simple “wear and tear” disease of cartilage and bone, but a whole organ disease, including several soft tissues such as the IPFP [32]. Accordingly, there is an increasing focus on systemic treatment approaches for OA, such as anti-inflammatory and anti-angiogenic medication [33]. In future trials, it will be a prerequisite to identify OA subtypes, in which advanced MR imaging, such as DCE-MRI, could potentially play a major role.

The different results for PFP found in this study are not consistent with current insights in PFP, which is supposed to be a precursor of OA [34, 35]. A possible explanation might be that tissue homeostasis is not yet as disturbed in PFP and inflammatory cytokines are not yet released. Thus, even though T2 hyperintense IPFP regions appear identically on unenhanced T2-weighted fat-saturated MR images in OA and PFP patients as well as healthy controls, the results of our DCE-MRI analysis show that there are different degrees of perfusion within the IPFP of controls, PFP patients, and OA patients, which may point towards different pathophysiologies. This knowledge will help the practicing radiologist who is confronted with an increased application of sensitive knee MRI to appraise these lesions in the context of the patient’s age and concurrent abnormalities.

In this study, the IPFP was quantitatively analyzed by T2 mapping and DCE-MRI in order to investigate the pathophysiology of T2 signal alterations in the IPFP within OA and PFP. The single prior study that applied DCE-MRI to investigate the IPFP by Ballegaard et al [23] used a different approach in the definition of the region of interest, as they focused on the entire IPFP in 3D rather than T2_FS_-hyperintensities within the fat pad. Furthermore, only obese patients with knee OA were included and a heuristic DCE-MRI analysis method without pharmacokinetic modeling was performed.

A strength of the current study is the quantitative assessment of DCE-MRI perfusion values, which offers more robust parameters that directly represent the microvasculature physiology, in contrast to semi-quantitative analysis. Furthermore, the inclusion of different patient groups from two studies offered the possibility to determine the nature of T2_FS_-hyperintense IPFP regions across different disease entities, one of which (PFP) has been suggested as a precursor to the each other (OA). We were able to directly compare the results of the quantitative DCE-MRI analysis from both studies because the exact same MRI scanner was used with identical scan and image post processing for both studies. Additionally, statistical analyses were performed within subjects of each subgroup, and thus possible differences in confounding variables between the subgroups will not have influenced our results.

A potential limitation was that no B1+ inhomogeneity assessment and T1 correction was possible, due to the lack of B1+ or pre-contrast T1 map. A fixed T1(0) value of 1443 ms (standard value of the DCE Tool used in Horos) was used instead. We expect that differences that may have arisen as a result of ignoring region T1 variability will not change the outcome of this study, as the observed differences in perfusion were large and substantially larger than any differences that we would expect due to T1 variability. Furthermore, we used a dedicated transmit/receive knee coil with relatively homogeneous B1 field. At the time of the MR acquisitions, linear gadolinium contrast agents, like gadopentetate dimeglumine, were commonly in use. Since then, these have been withdrawn from the EU market and have been replaced by alternatives that carry less risk for nephrogenic systemic fibrosis. As the perfusion kinetics of these alternatives are similar, we expect our results to be relevant for the newer generation contrast agents as well. Another limitation is that the OA group comprised patients referred for knee arthroplasty because of end-stage clinical OA, although the radiographic OA severity ranged from KL grade 2 to 4, with grade 4 relatively underrepresented. Furthermore, ROIs were drawn on one slice only. Finally, T2_FS_-hyperintense lesions were found only in 43 subjects, and the OA group was relatively small. However, all these subjects underwent an extensive MRI protocol including the administration of an intravenous contrast agent. In future research, it would be interesting to examine the perfusion of T2_FS_ hyperintense lesions in a population with a wider range of clinical OA severity, to evaluate the diagnostic value of T2_FS_-hyperintense lesions and their perfusion characteristics in classifying patients with unknown OA status, and to study the relationship of perfusion parameters with clinical symptoms.

In conclusion, T2_FS_-hyperintense regions of the IPFP demonstrated higher quantitative DCE-MRI blood perfusion parameters compared to adjacent tissue with normal signal intensity in patients with knee OA, but not in patients with PFP and healthy control subjects. This suggests different pathophysiology of IPFP T2_FS_-hyperintense regions across patient subgroups, in which an inflammatory pathogenesis is only present in OA.

## Abbreviations

AIF: Arterial input function
BMI: Body mass index
DCE-MRI: Dynamic contrast-enhanced MRI
FOV: Field-of-view
FSE: Fast spin echo
FSPGR: Fast spoiled gradient echo
IPFP: Infrapatellar fat pad or Hoffa’s fat pad
IQR: Interquartile range
KL: Kellgren and Lawrence
KOOS: Knee Injury and Osteoarthritis Outcome Score
MOAKS: MRI Osteoarthritis Knee Score
MRI: Magnetic resonance imaging
OA: Osteoarthritis
PFP: Patellofemoral pain
ROI: Region of Interest
SD: Standard deviation
